# 3D Printing and Shaping Polymers, Composites, and Nanocomposites: A Review

**DOI:** 10.3390/polym14010180

**Published:** 2022-01-03

**Authors:** M. N. M. Azlin, R. A. Ilyas, M. Y. M. Zuhri, S. M. Sapuan, M. M. Harussani, Shubham Sharma, A. H. Nordin, N. M. Nurazzi, A. N. Afiqah

**Affiliations:** 1 Department of Textile Technology, School of Industrial Technology, Universiti Teknologi MARA, Cawangan Negeri Sembilan, Kampus Kuala Pilah, Kuala Pilah 72000, Malaysia; 2 Institute of Tropical Forestry and Forest Products, Universiti Putra Malaysia, Serdang 43400, Malaysia; 3 Faculty of Engineering, School of Chemical and Energy Engineering, Universiti Teknologi Malaysia (UTM), Johor Bahru 81310, Malaysia; abuhassannordin@gmail.com; 4 Centre for Advanced Composite Materials (CACM), Universiti Teknologi Malaysia (UTM), Johor Bahru 81310, Malaysia; 5 Advanced Engineering Materials and Composites Research Centre (AEMC), Department of Mechanical and Manufacturing Engineering, Universiti Putra Malaysia, Serdang 43400, Malaysia; mmharussani17@gmail.com (M.M.H.); afiqahdnn@gmail.com (A.N.A.); 6 Department of Mechanical Engineering, IK Gujral Punjab Technical University, Main Campus, Kapurthala 144603, India; shubham543sharma@gmail.com; 7 Department of Mechanical Engineering, University Centre for Research and Development and Chandigarh Universiti, Pubjab 140413, India; 8 Centre for Defence Foundation Studies, Universiti Pertahanan Nasional Malaysia (UPNM), Kem Perdana Sungai Besi, Kuala Lumpur 57000, Malaysia; mohd.nurazzi@gmail.com

**Keywords:** 3D printing, 4D printing, additive manufacturing, biocomposite, industry revolution, polymer

## Abstract

Sustainable technologies are vital due to the efforts of researchers and investors who have allocated significant amounts of money and time to their development. Nowadays, 3D printing has been accepted by the main industry players, since its first establishment almost 30 years ago. It is obvious that almost every industry is related to technology, which proves that technology has a bright future. Many studies have shown that technologies have changed the methods for developing particular products. Three-dimensional printing has evolved tremendously, and currently, many new types of 3D printing machines have been introduced. In this paper, we describe the historical development of 3D printing technology including its process, types of printing, and applications on polymer materials.

## 1. Introduction

Most manufacturing sectors have constructed a product by using subtractive manufacturing and carving out the design on a solid block of material. However, recently, those sectors have adopted 3D printing technology in their manufacturing process. Three-dimensional printing technology has surpassed other manufacturing methods as it has been used in various areas on a myriad of different applications. It is also known as a method for additive manufacturing, whereby more intricate interior design modeling can be produced since it uses a layer-by-layer method. In addition, this technology of rapid prototyping can produce a prototype in a short time period, subject to the complexity of the design. Moreover, the technology allows manufacturers to test and evaluate 3D models, before the end-products are produced, by using computer-aided design (CAD) software. The 3D model is constructed by the machine [1]. In this paper, we aim to review the 3D and 4D printing processes and applications of this technology in the manufacturing industry. Initially, we describe the historical development of 3D printing, advantages and disadvantages of 3D printing, types of 3D printing, the process of 3D printing, and the development from 3D printing to 4D printing. Then, we also discuss the novel materials used in 3D and 4D printing applications.

## 2. Background

### Historical Development of 3D Printing

The earliest research on the use of photopolymers for creating 3D objects was conducted in the 1960s, at the Battelle Memorial Institute in Ohio. The study used two laser beams that intersected at different wavelengths to polymerize resin. Wyn Swainson developed the first pattern regarding 3D printing by using photochemical machining that also involved dual laser beams, and applied for a patent in 1971 [2]. Later, he also formed the Formigraphic Engine Company in California; however, the technology was not commercialized and made available for manufacturers at that time [3]. In the same period, solid photography was invented by Dynell Electronics Corporation in the late 1970s. The technology used a laser to cut cross-sections based on a model, and then stacked them to form objects [4].

The use of 3D printing has been increasing since the 1980s. For example, Charles Hull printed a three-dimensional object for the first time in 1983. Three-dimensional systems have been created using the technique of stereolithography (SLA) as well as the first program for virtualization. The invention has gained the attention of people in the field of architecture because it has increased the potential for direct manufacturing of parts [5]. In 1984, he obtained a patent for 3D printing and later co-founded 3D Systems, Inc., which is one of the leading companies in 3D technology. The company managed to commercialize the system with a printer and developed a new file format called STL (stereolithography) which is a technology that can detail the surface geometry of 3D objects. Charles Hull also worked with Lisa Crump through a new company called Stratasys to develop fused deposition modeling, in the late 1980s, and subsequently obtained several patents [6].

In addition, Hans Langer from Germany formed Electro-Optical Systems (EOS), in 1989, which produces 3D parts using metal laser sintering based on the model from a computer [7]. All dual transfer mode (DTM) patents related to laser sintering were acquired by EOS in 2004 [8]. Carnegie Mellon and Stanford, in the 1990s, suggested a new method of additive manufacturing using spraying [9] and microcasting [10] of materials.

Furthermore, the 3D printing industry was divided into two areas during the mid-1990s, i.e., medical equipment (highly engineered complex parts) and user-friendly printers for cost-effective prototype improvement concepts. However, by the end of the 1990s, only three original companies remained, i.e., EOS, 3D Systems, Inc., and Stratasys [4]. Three-dimensional printing technology was commercially accepted by most industries by the early 2000s and turned out to be a reliable technology to produce end-products. Adrian Bowyer, at the University of Bath, initiated a project called the RepRap project. The project created a 3D printer that could reproduce itself through this open-source project [11].

## 3. Advantages and Disadvantages of 3D Printing Production

The emergence of 3D printing technology has transformed most manufacturing industries into new standard levels. Many factors such as time, cost, and design work have been affected and have evolved tremendously. The main advantage of 3D printing is the manufacturing cost which traditionally involves workers and several machines for a product to be completed [12], whereas 3D printing offers a single-step manufacturing process, which reduces the cost and time for a product to be completed. It has also become a popular choice due to rapid prototyping that can produce flexible and more intricate designs with higher flexibility [13]. Waste during the process has also been minimized as compared with traditional manufacturing, since 3D printing products are produced without molds, and therefore less material and reduced waste. Almost all products, from basic to advanced applications, can be manufactured using the technology. The manufacturing works have also become simpler, involving computerized software and a 3D printer, thus, making it more accessible to a broader range of people [14].

However, the technology also has its drawbacks, mainly in terms of limited material and product build size. Different melting characteristics of polymers have limited the usage of certain polymers since the polymer temperature needs to be carefully controlled during 3D printing. Currently, a 3D printer has a small/limited printing chamber, and bigger products have to be printed in separate parts. Consequently, the cost of the products will increase since additional assembling processes are needed. The elimination of several processes in 3D printing has placed low-skill manufacturing jobs at risk due to automation by 3D printers. Moreover, some 3D printers with lower printer tolerance can have product or design accuracy issues during printing and need to be serviced after being used; post-processing after use indirectly increases production costs. Finally, copyright issues may occur due to the high accessibility of the technology, and therefore people tend to produce similar products that are very difficult to distinguish. Table 1 shows the summary of advantages and disadvantages of 3D printing.

## 4. Terminology of Additive Manufacturing According to ISO/ASTM 52900

Additive manufacturing is the general term for those technologies that, based on a geometrical representation, create physical objects by successive addition of material. These technologies are presently used for various applications in the engineering industry as well as other areas of society, such as medicine, education, architecture, cartography, toys, and entertainment. During the development of additive manufacturing technology, there have been numerous different terms and definitions in use, often with reference to specific application areas and trademarks. This is often ambiguous and confusing which hampers communication, and therefore wider application of this technology. International standards have been developed by ISO/TC 261 and ASTM F42 in close cooperation on the basis of a partnership agreement between ISO and ASTM International with the aim to create a common set of ISO/ASTM standards for additive manufacturing. Table 2 shows the general terms and definitions for additive manufacturing according to ISO/ASTM 52900.

## 5. 3D Printing Process

**Figure 1:**
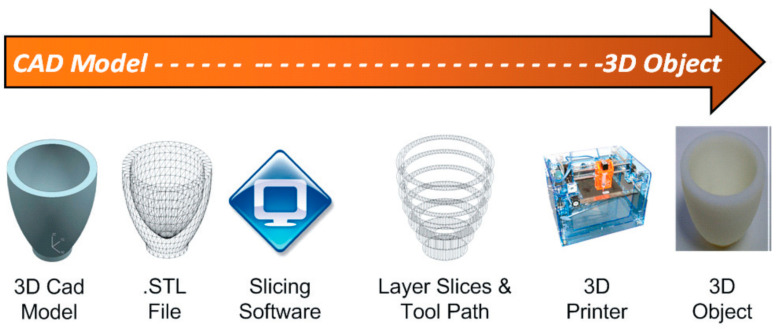
The 3D printing process (adapted with permission from reference [18]).

**Figure 2:**
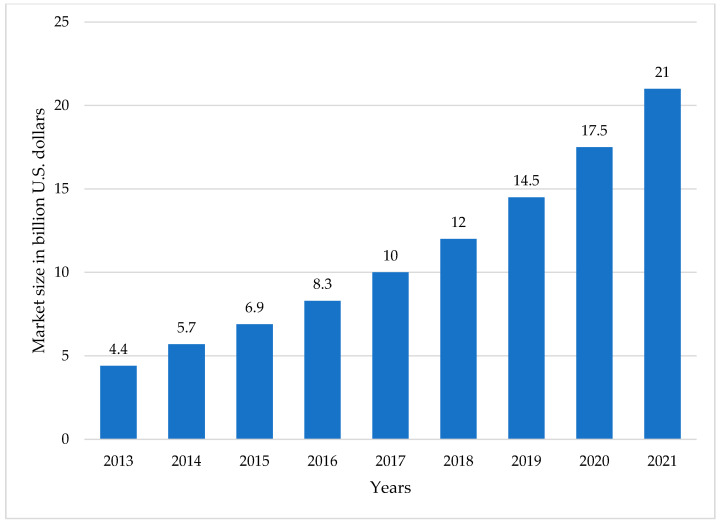
3D printing market size worldwide, from 2013 to 2021.

The 3D printing process started by developing an object model in 3D form by using CAD software or reverse engineering technique using a 3D scanner to scan the finished products. The 3D software cross-sectionally cuts the layers of the model, and then transfers a file from the computer to the printer. Then, the printer creates layers of objects through the selective placement of material. The process is the same as the working principle of an inkjet printer that goes front and back over the page to add layers of material on the upper parts of each other until the objects are obtained [15]. There are three main steps in the 3D printing process which are modeling, printing, and finishing (Figure 1). The popularity of the 3D printer has continued to grow due to the development of 3D modeling technologies and techniques [16] (Figure 2). The global 3D printing market size was valued at 14.5 billion USD in 2019 and is expected to expand at a CAGR exceeding 14% from 2020 to 2027, according to Grand View Research’s latest report [17]. In addition, 3D printing and related technologies have evolved continuously in line with the intensive R&D activities undertaken and the aggressive investments made by private and public sectors. Government funding and encouraging initiatives offered in developing economies have prompted manufacturers to pursue improvements in technology and the adoption of new technologies.

Further key findings from the report according to Grand View Research’s latest report [17] suggest:

- The capability of 3D printing technology to provide precise and quick prototypes while also reducing time to market is gaining popularity.
- The market is expected to increase considerably as 3D printers become more widely used in the medical, transportation, and electronics industries.
- Revenue for desktop 3D printers is projected to rise over the market growth, as 3D printing is becoming more popular among enthusiasts for personal, home, and recreational use, as well as in educational institutions for training reasons.
- In 2019, the prototyping segment led the market, and it is anticipated to grow to more than 50% by 2027.
- The polymer sector accounted for over half of the total market share. In the next seven years, however, the metal category is anticipated to dominate the market. This might be due to the growing demand for metal 3D printing from industries including automotive, aviation, and military.
- Over the projection period, the desktop 3D printing industry is likely to embrace the 3DP technology rapidly and likely will be further divided into educational purposes, fashion and jewelry items, dentistry, nutrition, and other categories.
- The Asian Pacific 3D printing economy, which is becoming a manufacturing powerhouse for several industrial sectors, is projected to grow significantly as urbanization drives infrastructural needs and encourages verticals such as automobiles, consumer electronics, engineering services, and medicare to adopt 3D printing, particularly in China, Japan, and South Korea.
- Stratasys Ltd., 3D Systems, Inc., 3D Ceram, GE Additive, HP Inc., Tiertime, EnvisionTec, Inc., and Dassault Systemes are some of the market’s major competitors.

## 6. Types of 3D Printing

**Figure 3:**
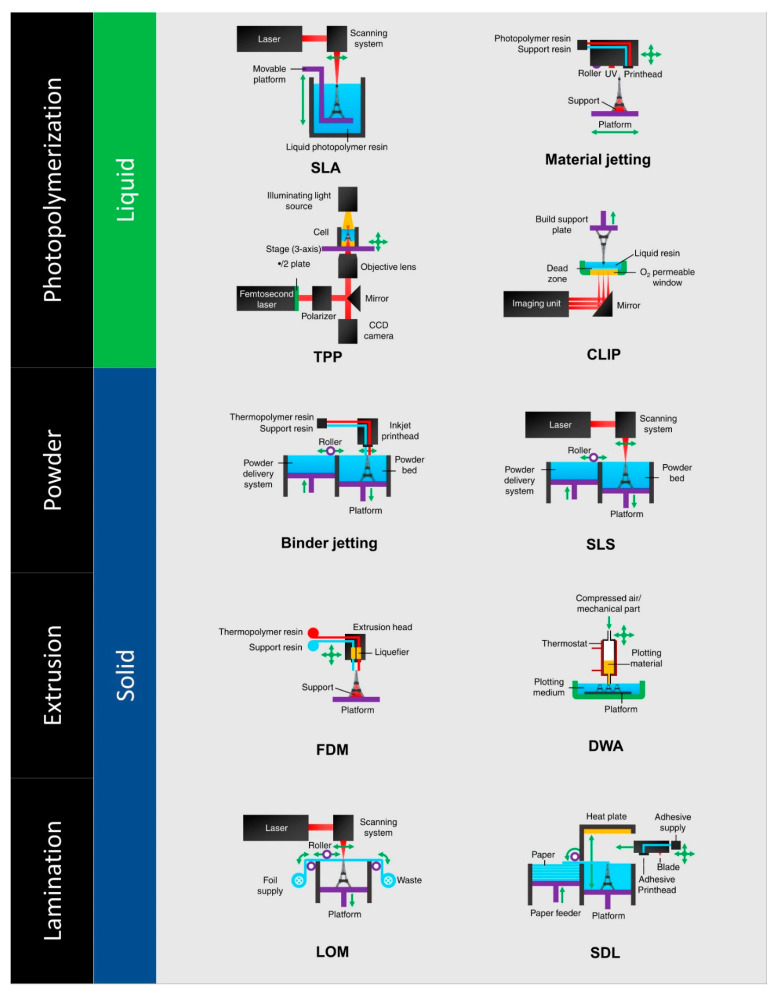
Types of 3D printers (adapted with permission from reference [20]).

There are several types of 3D printing machines available that are used in the manufacturing field (Figure 3). All of the machines can be differentiated based on their technology [19]. Printers have been developed based on suitability for the material to be printed and some of them are namely: fused deposition modeling (FDM), laminated object manufacturing (LOM), digital light processing (DLP), stereolithography (SLA), selective laser melting (SLM), selective laser sintering (SLS), electronic beam melting (EBM), binder jetting (BJ), and material jetting (MJ) [19].

### 6.1. Fused Deposition Modeling (FDM)

**Figure 4:**
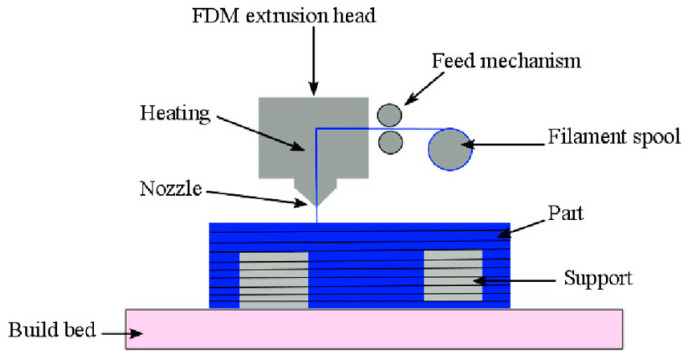
Fused deposition modeling (FDM) process (adapted with permission from reference [24]).

FDM is the most popular technology for 3D printing based on Wohler’s Report from Stratasys, Inc (Figure 4). The main elements of the system include the feeding mechanism, print head, gantry, build surface, and liquefier [21,22,23].

**Figure 5:**
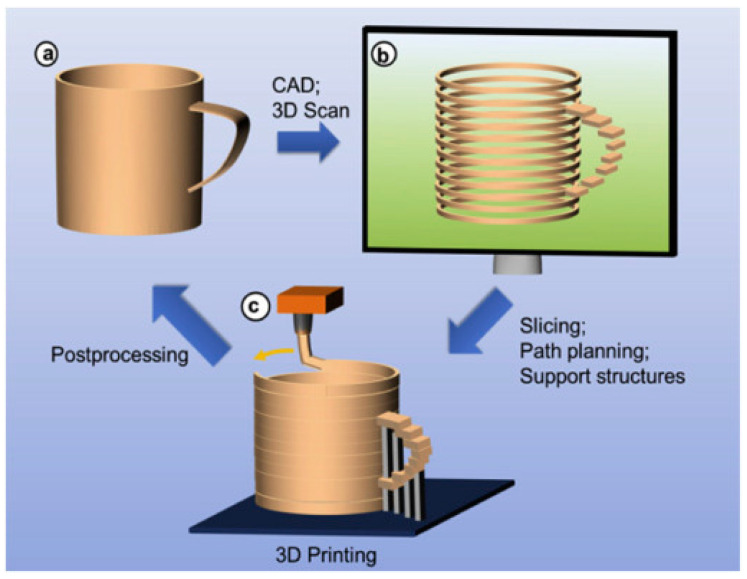
Depicting the route in 3D printing to obtain a physical model from the digital model (adapted with permission from reference [26]).

Daminabo et al. [25] indicated that, while additive production (AM) technology has developed remarkably so far, there is still a significantly smaller range of printable and functional material systems that meets the needs of a wide range of industries, including healthcare, manufacture, packaging, aerospace, and the automobile industry. Furthermore, the increasing need for sustainable development has directed attention to extrusion-based technologies (fused deposition models and direct ink writes) because of their scalability, cost effectiveness, and processability of a wider variety of materials, as demonstrated in Figure 5. The authors then identify the new materials and current research efforts that can assist the development of functional/multifunctional (4D) components and products based on extrusion technologies in a sustained and sustainable way.

**Figure 6:**
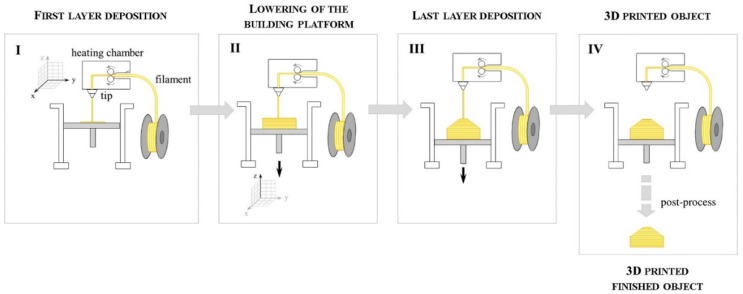
Outline of FDM production cycle (adapted with permission from reference [27]).

Melocchi et al. [27] explored the feasibility of fused deposition modeling (FDM) 3D printing in the manufacturing of capsular devices for oral pulsatile release based on a swellable/erodible polymer (hydroxypropyl cellulose, HPC). The production cycle of FDM is shown in Figure 6. This involved an experimental assessment of manufacturing hollow objects through FDM and HPC filaments (not commercially accessible) through thermal melt extrusion (HME). In addition, it was necessary to confront the setup of suitable computer-aided design files. The HME and FDM processing used a two-screw extruder fitted with a rod-formed die and a deliberately built sweeping/calibration mechanism, as well as a MakerBot Replicator 2 3D printer. Bodies and caps were developed with suitable physical and technological characteristics. This proved the ability to make capsular devices for oral pulsation of FDM 3D printing using purposefully produced HPC filaments and evaluated FDM’s real-time prototyping capabilities.

**Figure 7:**
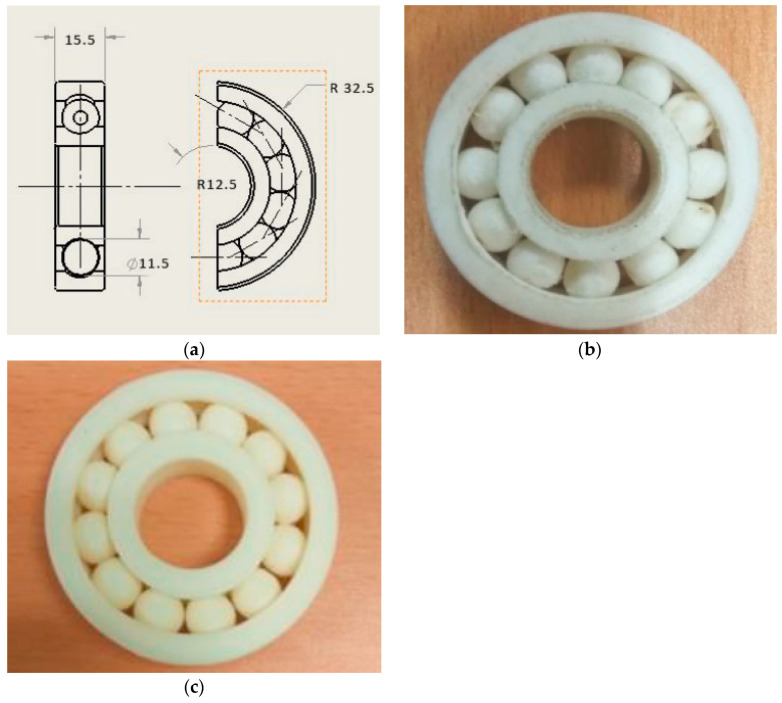
(**a**) 2D drawing of the assembly (dimensions in mm) and 3D-printed bearing using (**b**) Nylon 66 and (**c**) ABS (adapted with permission from reference [28]).

Harikrishnan and Soundarapandian [28] thoroughly researched the construction of layer-by-layer components, offering a lot of flexibility and freedom in the design and production of additives. The use of this technology has shifted over the past several years from basic prototyping of functioning components to manufacturing. However, most functioning components are assembly components. In this study, an attempt was made to construct a mobile component ball bearing that would ordinarily need installation in a single step. A simple desktop fused deposition modeling (FDM) printer was used to print in different materials. PLA, ABS, and Nylon 66 were used to print the bearing, which is shown in Figure 7b,c. Surface characteristics such as area surface roughness and Shore D hardness were measured and compared with the materials produced. FDM was shown to be able to manufacture complex moving parts in one step.

**Figure 8:**
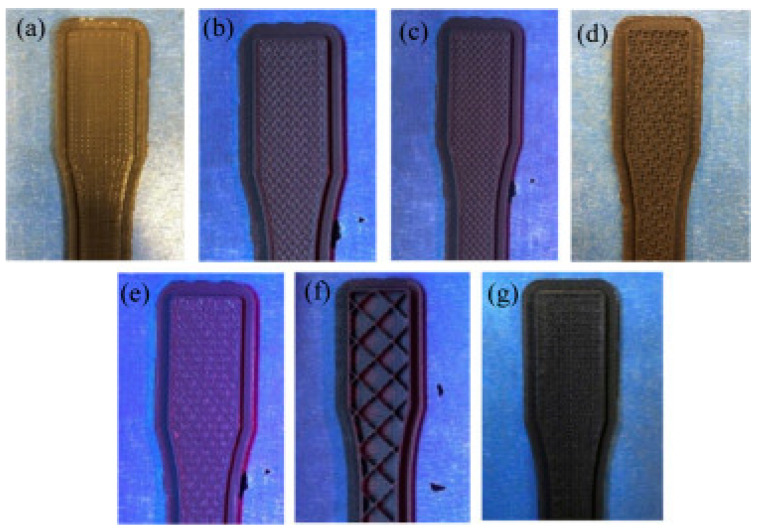
3D-printed samples with different infill patterns: (**a**) Linear; (**b**) hexagonal; (**c**) moroccanstar; (**d**) catfill; (**e**) sharkfill; (**f**) diamond; (**g**) Hilbert (adapted with permission from reference [29]).

Abeykoon et al. [29] investigated 3D printing, a common technology for producing 3D digital solid structures. The correct selection of printing settings is important in order to generate high-quality 3D-printed components. The focus of this study was on the characteristics of 3D-impressed specimens with varied processing circumstances, such as infiltrations, density, fill speed, and diverse materials, including mechanical, thermal, and morphologic. The findings demonstrated that the Young’s modulus of the printed components increased with an increase in infill density. The strength of the samples printed was determined by their layer arrangement, shown in SEM images. It was also observed that 215 °C and 90 mm/s were the most optimal processing temperature and infill speed for pure PLA filament, respectively. The highest Young’s modulus, i.e., 2637.29 MPa, was obtained by 3D-printed carbon fiber reinforced PLA (CFR-PLA). This was because voids inside the matrix and layer gaps led to cracks in the specimens [30]. Different infill patterns of 3D-printed components which were utilized in this investigation are shown in Figure 8, with 100% infill, 90 mm/s, and 215 °C set nozzle. Overall, the linear filling pattern was the ideal pattern as analyzed by their mechanical and thermal properties. Haryati et al. [31] also conducted a study on the effect of chemically treated kenaf fiber on the mechanical and thermal properties of PLA composites prepared through fused deposition modeling (FDM). The treatment of kenaf fiber with a NaOH concentration of 6% for 24 h, followed by a chemical treatment of silane coupling agent with three different concentrations, i.e., 0.5%, 1%, and 2%, respectively, for 3 h, was performed to modify the surface characterization of the natural fiber. The samples were mixed and extruded to produce biodegradable filament biocomposites. Then, the sample was extruded using a Flashforge 3D printing nozzle with the temperature set to 210 °C and bed temperature at 60 °C. The result shows that 1.0% silane concentration after being treated with a 6% alkali solution indicated an improvement in the interfacial bonding between two phases. This was because the removal of chemical content in the natural fiber itself, such as hemicellulose and lignin, resulted in the fiber surface becoming rougher.

### 6.2. Laminated Object Manufacturing (LOM)

**Figure 9:**
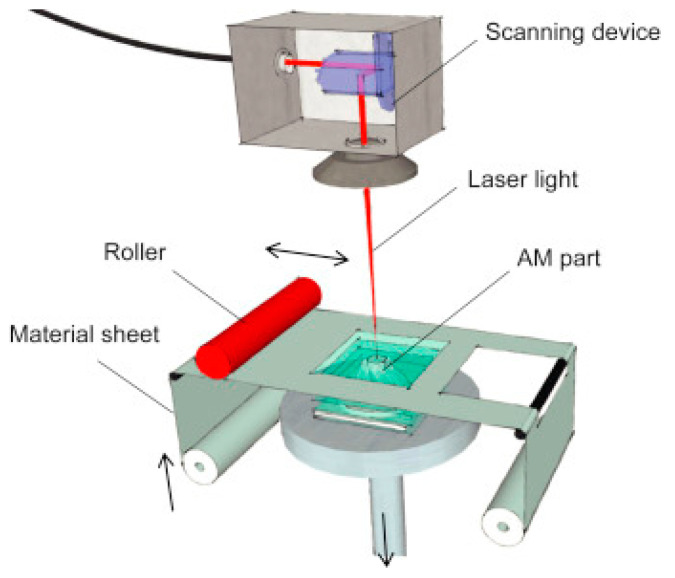
Laminated object manufacturing (LOM) process (adapted with permission from reference [33]).

Laminated object manufacturing (LOM) is a process to build an object by combining subtractive and additive techniques (Figure 9). The process binds several layer sheets of material, one on top of another using pressure, adhesive, and heat. After the binding process is completed, the product is cut using a laser, a knife, or can be further modified using machine drilling [18]. This technique is one of the high-speed prototype technologies used for creating 3D solids with a lamination process. The only characteristic of this technology is its ability to produce complex geometric components at lower production and operating costs. The LOM method was investigated by several researchers from the 1980s and produced promising results. The major aim of this study was to demonstrate the overall idea and operating principle of LOM and to examine the available paper, composite, and metal technologies [32].

**Figure 10:**
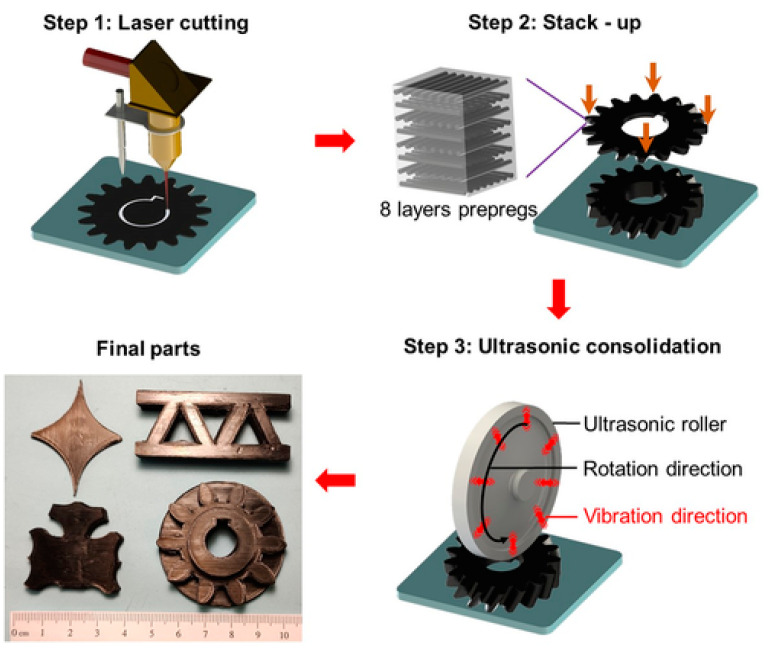
Schematic illustration of the ultrasonic-assisted laminated object manufacturing via AM method (adapted with permission from reference [34]).

Chang et al. [34] focused on the AM of continuous fiber-reinforced thermoplastic composites (CFRTPCs), which can produce innovative lightweight components with high durability at a relatively low cost. A new methodology for CFRTPC production was based on the new method of prepreg carbon fiber sheets, i.e., the manufacture of ultrasonic-assisted LOM, as shown in Figure 10. Prepreg sheets were initially cut in 2D form, followed by using an ultrasound vibration roller to consolidate every eight sheets in order to efficiently manufacture 3D composite components. Next, the additives were subjected to a hot press to further improve their mechanical properties. High tensile strength and tensile modulus of 1760 MPa and 106 GPa, respectively, were present in the unidirectional composite samples. The hot-pressed AM composite pieces have been used by the prepreg manufacturing process using typical production processes to determine mechanical characteristics. In general, there are tremendous potential applications in the aerospace and transportation industries for the suggested CFRTPC AM technique.

Olivier et al. [35] studied the best building orientation for components produced via the LOM technique to enhance their flexural performance. The results from previous studies demonstrated that components generated through LOM could resist greater deflections than components created by other production processes for layers. However, the relationship between building orientation and component flexural strength had not yet been evaluated. Four specimen types were produced for each type, utilizing various structural directions. In a machine with four charging points, the specimens were examined to assess their failure mechanism and to find the optimal building orientation towards bending loads. In terms of maximal load before failure, the optimal construction orientation was 45°. In addition, for each condition examined, a recurrent failure pattern was identified. The structural orientation was validated by affecting the mechanical characteristics of components as a significant LOM production parameter [36].

**Figure 11:**
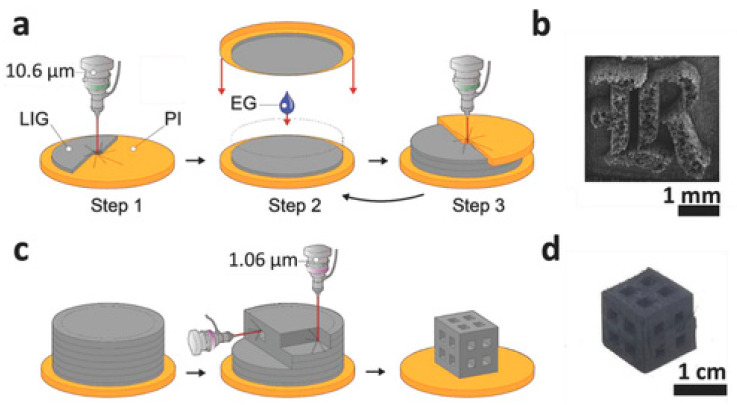
Laser-induced GFs are manufactured and processed: (**a**) Diagram of the LOM procedure; (**b**) milled LIG foam in a 3D “R” form. The LIG foam has a height of around 1 mm; (**c**) fiber laser milling process diagram; (**d**) LIG foam produced in three dimensions using a mix of LOM and fiber laser machining (adapted with permission from reference [37]).

Luong et al. [37] produced 3D laser-induced graphene (LIG) foams by producing layers of LIG by irradiating polyimide (PI) sheets, as illustrated in Figure 11. The PI to LIG conversion has been well described. To begin, layers of PI are irradiated to embed LIG into and atop a PI film. One of the layers serves as the GF’s foundation or basis. Then, the layers are layered on top of one another after being sprayed with ethylene glycol (EG). A laser-milling subtractive method is also created and illustrated here to further improve the 3D constructions. Various 3D graphene items are produced using a combination of both processes. Then, the sandwiched layers are lased, fusing the LIG sheets together, and this procedure is repeated to create macroscale LIG foams. The printed foam is dried in the environment at 200 °C to evaporate the leftover EG once the procedure is completed to the appropriate height. The LIG foams have high mechanical conductivity and are suitable for a variety of energy storage and electrical sensor applications [37].

### 6.3. Digital Light Processing (DLP) and Stereolithography (SLA)

**Figure 12:**
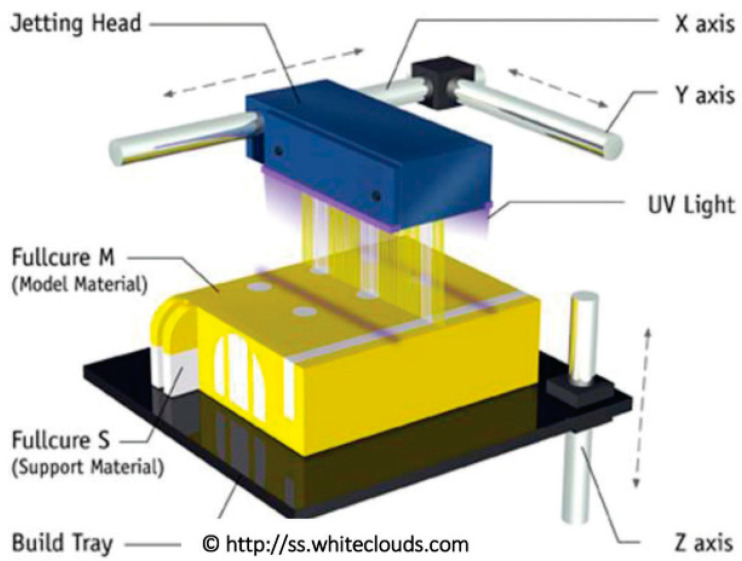
Digital light processing (DLP) process (adapted with permission from reference [39]).

The technology of 3D printing has gained significant research interest recently for directly manufacturing 3D components and structures for use in a variety of applications. Digital light processing (DLP) is a process to cure liquid resin to form 3D products using a digital micromirror device as a power source projector (Figure 12). DLP also uses light polymerization, which is almost the same method for stereolithography (SLA). In addition, DLP is a faster printing process, can produce objects with a higher resolution, and the thickness of the layer can be less than 30 microns [38].

According to Mu et al. [40], digital 3D printing technology based on processing light can be utilized in order to manufacture electrically conductive polymer nanocomposite where the film has been produced with a combination of photocurable resin and mixed nanotubes of carbon (MWCNTs). In order to achieve optimal conductivity and printing quality, MWCNT concentrations, as well as pressure factors, were examined. The results showed a maximum electrical conductivity of MWCNT loading in the resin matrix of 0.027 S/m under resin viscosity, allowing for good printing quality to be achieved by 0.3 wt.%. The impressed MWCNT nanocomposites can be employed as intelligent materials and constructions with stress sensitivity as well as form memory effect with electric conductivity [41,42]. DLP 3D printing for complex conductive structures has been done using printed conductor complex structures, such as a hollow capacitive sensor, electrically activated memory composites, and stretchy circuits.

**Figure 13:**
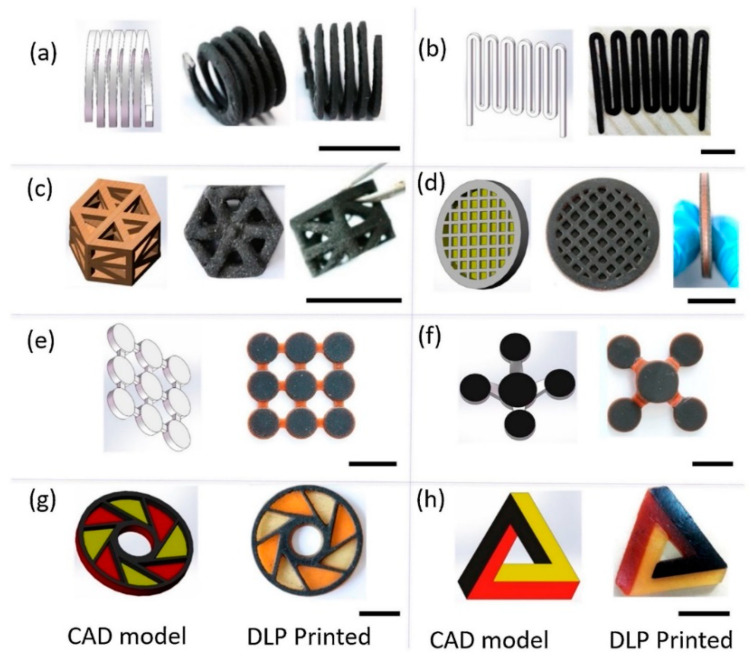
According to specified CAD models, various varieties of DLP 3D-printed conductive structures using MWCNT nanocomposite and immaculate resins. For printed items, the scale bar is 10 mm (adapted with permission from reference [40]).

Furthermore, mechanical testing has shown that the inclusion of MWCNT may somewhat raise the modular tensile stress and slightly reduce the ultimate tensile stress, showing that additional functionality is not achieved at the expense of mechanical qualities being compromised. Figure 13 depicts several types of DLP 3D-printed conductive structures made with MWCNT nanocomposite and pristine resins, as created using CAD models on the left side of the produced objects; only MWCNT nanocomposite was used to print the items in Figure 13a–c (Figure 13a, hang structured spring; Figure 13b, planar wave shape resistor; and Figure 13c, hollow structure truss). The structures in Figure 13d–f were printed with two types of materials, i.e., MWCNT nanocomposite and pristine resin (Figure 13d, capacitor with hollow structure; Figure 13e, capacitor array; and Figure 13f, capacitors at various heights). Figure 13g,h shows the structures printed using three different materials, i.e., partially conductive structures printed by MWCNT nanocomposite, pristine orange resin, and pristine yellow resin [40].

**Figure 14:**
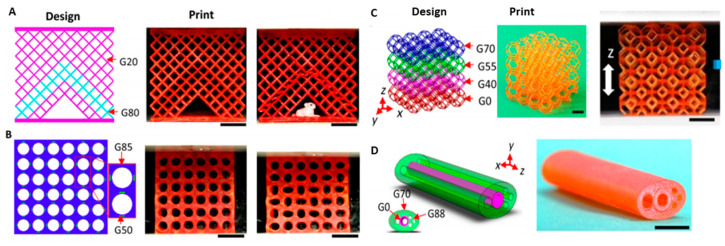
Design and print of graded metamaterial via g-DLP for multifunctional applications, including 2D lattice and cellular metamaterial for: (**A**) Controlled buckling; (**B**) pattern transformation; (**C**) 3D lattice metamaterial; (**D**) the design and print part of a limb-mimic structure. Scale bars, 1 cm. (Adapted with permission from reference [45]).

According to Cortes et al. [43], 3D printing was widely utilized to improve the mechanical and stress sensing capacities of carbon nanotube (CNT)-based components. The composition of the CNT was examined as well as post-curing treatment effects. The effects on mechanical characteristics, after the treatment, were increased Young’s modulus and increased glass transition temperatures, but their effect was not as relevant for electrical properties. In addition, the strain sensing experiments demonstrated a linear response to applied strain, with greater sensitivity values when the content of the CNT was decreased due to a higher inter-particular distance [44]. As a result, the nanocomposites possessed enhanced gauge factor values as well as a better linear response, demonstrating their suitability for health monitoring applications. In order to enhance the mechanical characteristics of 3D-printed prototypes, Kuang et al. [45] explored a single-vat grayscale digital light processing (g-DLP) 3D printing technology employing grayscale light patterns and a two-stage curing ink. Figure 14 shows the design and print part of 2D and 3D lattice and cellular metamaterial.

**Figure 15:**
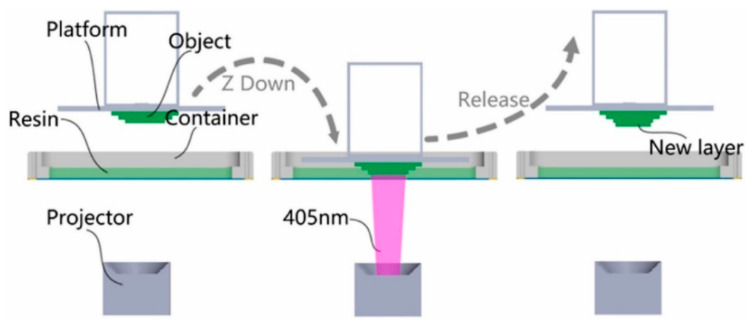
3D printing process from the bottom to the top (adapted with permission from reference [46]).

Zhao et al. [46] investigated the impact of adding fiber filler on plastic strengthening, which is worthwhile in 3D printing technology for digital light processing (DLP). This study assessed the application of stereolithography with resin micro-sized poly(p-benzoyl-p-phenylenediamine) (Kevlar). The Kevlar fiber and resin combination has an appropriate viscosity. The resin was hardened in the *z*-axis direction layer-by-layer through the projector beam (405 nm) using the procedure shown in Figure 15. The results showed that once the Kevlar content reached 7 wt.%, the subject could still be printed, and proved that Kevlar fibers have potential applications for DLP 3D printers as compared with other techniques that use Kevlar fiber.

To improve the mechanical characteristics of cured components, Xie and He et al. [47] manufactured a nanocomposite of graphene oxide and resin using a liquid crystal display (LCD) mask light curing technique. Excess graphene oxide (0.5 wt.%) was reported to block light and failed to print. This means that transparency is a crucial characteristic for composite 3D printing. Li et al. [48] studied cellulose nanocrystals (CNC) which have received significant attention due to their high Young’s modulus, high strength, biocompatibility, and renewability. These characteristics make them excellent for a polymer composite strengthening phase [49,50]. However, standard composite processing procedures have restrictions when composites with diverse forms are manufactured efficiently. The digital light processing (DLP) 3D printing approach was utilized to fabricate CNC reinforced poly(ethylene glycol) diacrylate (PEGDA) glycerol composites with great fidelity. The mechanical tests showed that CNC integration had enhanced the mechanical characteristics of DLP 3D-printed composites. DLP 3D printing may also be used to promote the use of cellulosic materials for biological applications.

**Figure 16:**
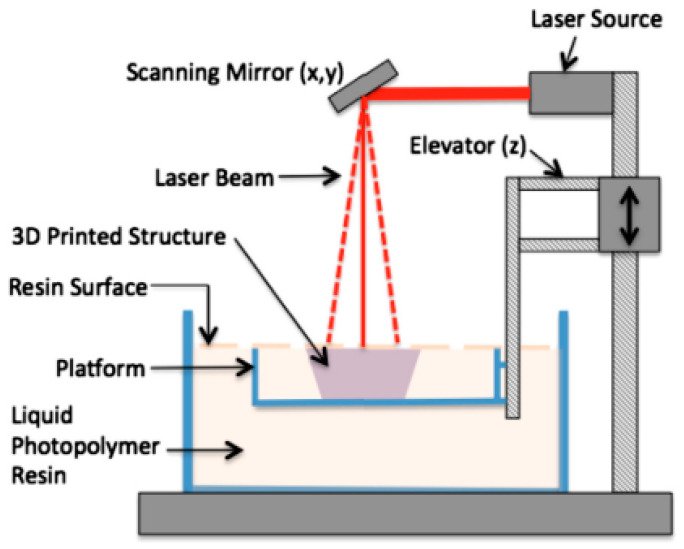
Components of a stereolithography system.

The DLP and SLA technologies are very similar, as they are both classified as additive manufacturing (AM) technologies by the American Section of the International Association for Testing Materials (ASTM). Nonetheless, SLA is the oldest 3D printing technology that is still being used today. Unlike DLP, the SLA 3D printer uses a laser beam to harden the polymer. Particularly, SLA uses a focused laser beam to scan the surface of each layer and provide the energy for polymerization (as shown in Figure 16), whereas DLP uses UV light from a projector. In addition, the laser beam of SLA moves from point to point tracing the geometry, while in DLP, the UV light source is stationary and cures the entire resin layer at once. As compared with DLP 3D printing, SLA 3D printing is more accurate and has better print quality because the resin is cured (hardened) from point to point.

### 6.4. Selective Laser Melting (SLM) and Selective Laser Sintering (SLS)

**Figure 17:**
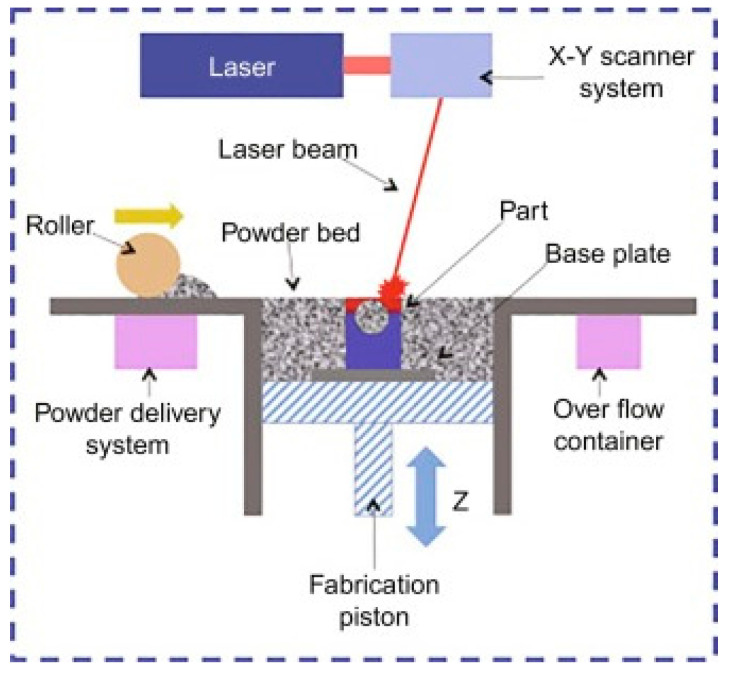
Selective laser melting (SLM) process (adapted with permission from reference [52]).

Selective laser melting (SLM) can produce high-precision, full-density, functional metal parts (Figure 17). The powder deposition method has been used in SLM that involves a coating mechanism to deposit a layer of powder onto the substrate plate and a powder reservoir. SLM technology can produce complicated metal parts using layer-by-layer melting with any geometry and joining of powder materials based on the CAD model from the computer. The powder particles are fused during the process, followed by solidification [51].

**Figure 18:**
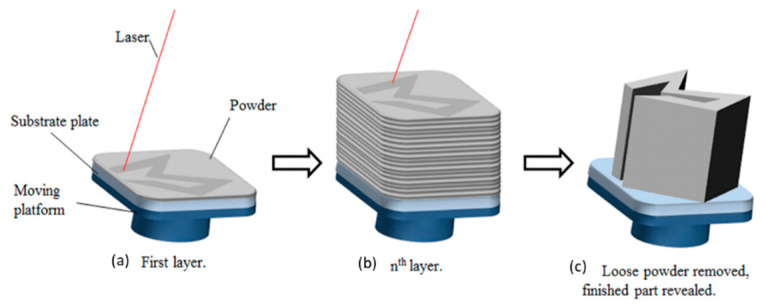
Recent SLM stage illustration comprised of three main steps: (**a**) Melting selective areas of the powder bed with a high-power laser; (**b**) repeating the process for successive layers; (**c**) cleaning the loose powder for the finishing stage. (Adapted with permission from reference [53].)

SLM is a specific rapid prototype, 3D printing, and AM technology for the purpose of melting and fusing metallic powders using a high-power laser. A component is made by fusing and melting powder selectively inside and among layers. The SLM process is also often called direct selective laser sintering, laser curing, and direct laser sintering, and has been proven to generate up to a relative density of 99.9% in close proximity to net-shaped components (Figure 18). This makes it possible to create functioning components of almost full density and offers practical cost advantages. SLM can deal with a variety of metallic materials, including copper, aluminum, and tungsten, owing to recent advancements in fiber optics and high-power lasers. Similarly, research prospects in SLM of ceramic and composite materials have opened up as a result of this [53].

**Figure 19:**
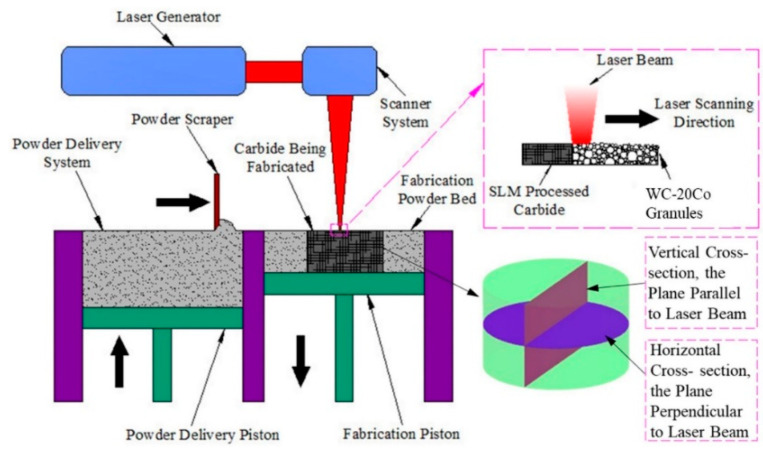
Schematic design of the SLM approach. On both the vertical (parallel to the laser beam) and horizontal (perpendicular to the laser beam) cross-sections of the SLM-treated carbides, microstructural analysis and hardness tests were performed (adapted with permission from reference [54]).

According to Chen et al. [54], cemented carbide (WC-Co) composite is hard to fabricate via a single-stage AM technique, including the SLM process shown in Figure 19. Thus, according to the literature, defect-free concrete carbide has never been properly manufactured by one-step SLM. SLM-controlled carbides were initially explored as crucial effects of feedstock carbide granulation shape on the microstructure. Without additional heat treatment, a crack-free WC-20Co cemented carbide with a high density was effectively synthesized utilizing one-step SLM. The density of the SLM-produced carbides was notably greater, albeit still unhelpful as compared with conventional liquid sintered carbides. The results demonstrated that spherical granules exhibited higher ultimate density as compared with non-spherical granules. The SLM technique led to the inhomogeneous and rapid growth of WC grain due to the uniform distribution of the temperature and to a varied period of time during the SLM process of materials in the liquid state.

Shen et al. [55] developed a selective laser melting (SLM) process for manufacturing a novel glass-fiber-reinforced glass (GRFG) composite material. Experiments were performed using a continuous-wave fiber laser to demonstrate this SLM method using S-glass borosilicate and fine soda powders with separate temperatures for glass transition, as shown in Figure 19. The fine glass granules became viscous during laser scanning. In order to avoid balling effect during SLM, a transparent borosilicate glass slide was placed on the top of the powder bed. The molten glass ran through the relatively solid fibers and did not fuse with them due to its relatively high glass transition temperature [56]. A compact GRFG composite formed with an unbroken, well-encompassed glass fiber high volume proportion when cooled.

**Figure 20:**
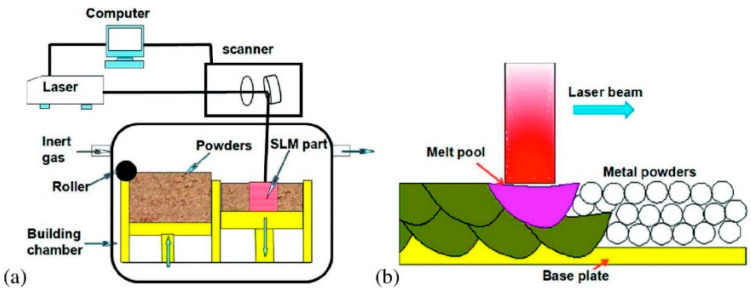
(**a**) Graphic representation of the SLM equipment; (**b**) the melt pool production method (adapted with permission from reference [57]).

Jue et al. [57] applied an SLM method to manufacture Al-based composites reinforced with Al2O3 particles, as illustrated in Figure 20. The SLM processing parameters had a significant influence on the densification behavior, morphology, mechanical, and wear performance. The findings demonstrated that the optimal speed of 550 mm/s resulted in a near complete dense composite component of 97%, which was facilitated by the trapping action of Al2O3 particles with the advancing interface in the molten pool. As a result, the Al2O3 particle dispersion homogeneity was improved. With a remarkably low coefficient of friction, the fully dense composite that was correctly produced demonstrated remarkable and enhanced wear performance.

**Figure 21:**
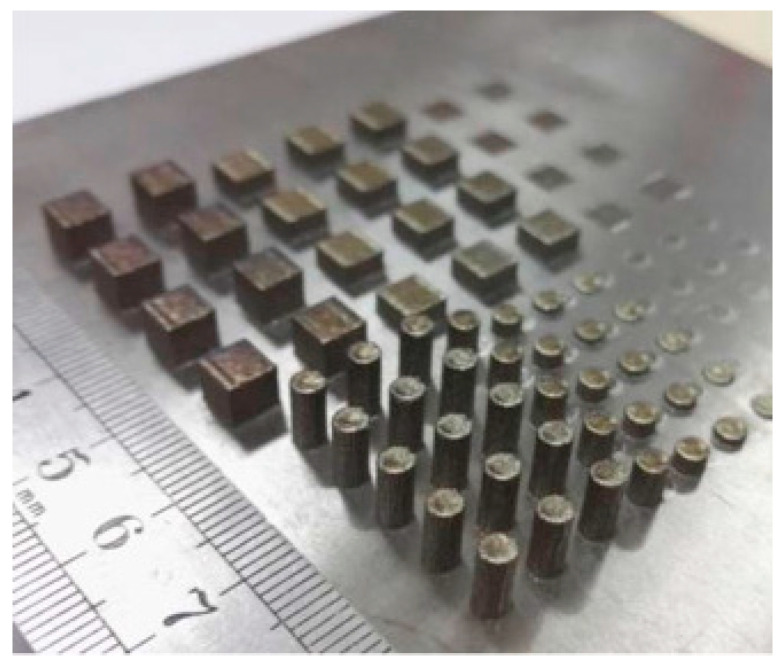
3D-printed Zr-based BMG samples (adapted with permission from reference [58]).

Three-dimensional printing of crack-free bulk metallic glasses remains to be a challenge due to their intrinsic brittleness and the generation of huge thermal stress during the selective laser melting. The selective laser melting process included the Zr55Cu30Ni5Al10 system and 3D printing, which is shown in Figure 21. There was evidence of a considerable (approximately 83%) portion of an amorphous phase of bulk metallic glassy composite and the successful production of small fragments of intermetallic compounds free from cracks. The strength of the 3D-printed metallic glassy composite was over 1500 MPa. A finite-element simulation revealed the mechanism of crystallization in heat-affected zones, demonstrating that low thermal stress reduced the probability of micro-crack formation and fracture toughness was important in crack suppression during the selective laser melting process [58].

**Figure 22:**
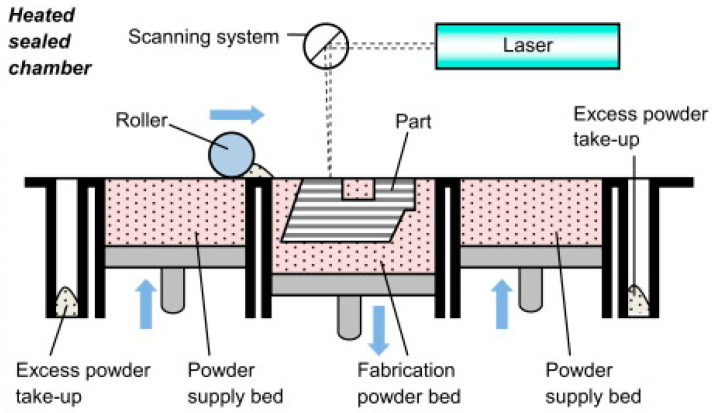
Selective laser sintering (SLS) process (adapted with permission from reference [59]).

Selective laser sintering (SLS) (Figure 22) also uses a high-power laser similar to selective laser melting (SLM), which uses power in the form of a high energy laser beam [18]. The difference is that the SLM method goes further, until fully melted powder is achieved, which means the powder is homogeneously melted instead of just fused. Furthermore, utilizing composite powders as feeding materials, AM approaches such as SLS offer techniques for creating 3D complex components with acceptable mechanical, electrical, and thermal characteristics.

**Figure 23:**
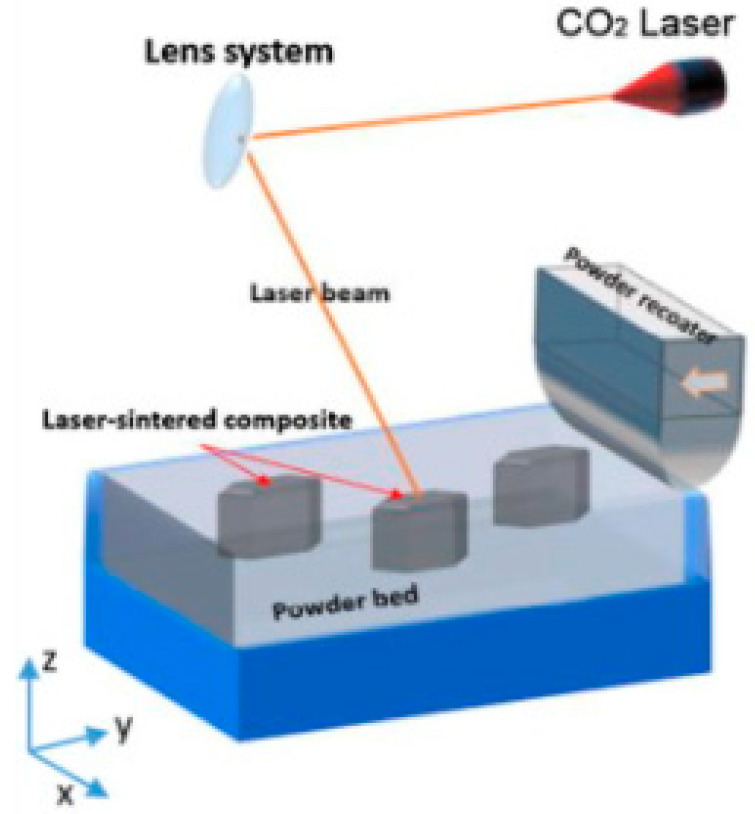
Illustrations of the SLS system (adapted with permission from reference [60]).

According to Yuan et al. [60], the SLS method provides a novel method for fabricating carbon nanotube (CNT) composite powders, see Figure 23. This method may be a more efficient way to produce CNT/polymer composites with electrically conductive segregated structures as compared with the common hot-compression technique. Furthermore, at a restricted loading range of CNTs of 1 wt.%, the SLS-derived composites show considerable increases in electrical conductivity, up to anti-static and conductive levels, validating the applications in vehicles and aircraft. The process-structure-property linkages are further examined in order to better understand the different processes that result in microstructures, as well as the underlying mechanisms that control thermal and electrical performance.

**Figure 24:**
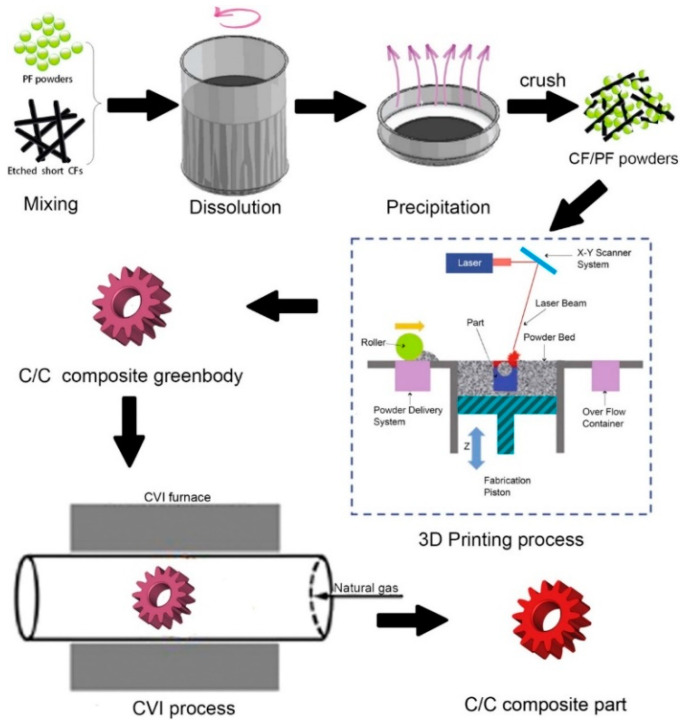
Overview of preparing carbon/carbon composites complex part (adapted with permission from reference [61]).

To produce complex carbon/carbon (C/C) composite components, as illustrated in Figure 24, a novel 3D printing process is designed. Combining selective laser sintering and the chemical vapor infiltration-thermal treatment technique yields C/C composites with excellent mechanical performance. It is possible to make C/C composites with a maximum density of 1.5 g/cm3 and a bending strength of 100 MPa. The computer-aided design technique is used to accurately construct complex C/C composite parts with a minimum thickness of 0.5 mm. This newly discovered 3D printing process can be used to make complex C/C composite items with excellent precision and mechanical performance [61]. Using self-made carbon nanotubes (CNTs) wrapped TPU powders, Li et al. [62] used the SLS method to create a flexible thermoplastic polyurethane (TPU) conductor. SLS printing is a unique approach to build segregated conductive networks of CNTs in a polymer matrix since it is a shear-free and free-flowing method that does not need compacting. With a percolation threshold of 0.2 wt.%, the electrical conductivity of the SLS processed TPU/CNTs composite was seven orders of magnitude greater than that of the traditional injection molded TPU/CNTs composites with the same CNTs content. Even after 1000 cycles of bending, the electrical resistance of the 3D-printed TPU/CNTs specimen maintained a relatively constant value. This method can readily produce flexible conductive TPU/CNT composites with complex topologies and forms, such as porous piezoresistors [62].

Espera Jr. et al. [63] stated that the SLS method was ideal for mass production of mechanically strong sintered components using large volumes of powder materials. This study demonstrated a simple method for mixing polyamide-12 (PA12) and carbon black (CB) powders for use in SLS. The study looked into the changes in the 3D-printed material’s mixing consistency, mechanical properties, and thermal stability. The quantity of CB in the sintered parts generated by the efficient separate grains mixing technique was associated with bulk resistivity, demonstrating the consistency of carbon content in the sintered parts. SLS was used to create 3D-printed components reinforced with CB. Mechanical characteristics decreased at concentrations larger than 3 wt.% due to CB particles interfering with physical interaction between PA12 particles, reducing the effectiveness of the sintering process. The CB/PA12 sintered components had stronger thermal stability than the clean PA12 sintered parts, resulting in higher degradation temperatures. As a result, this study effectively showed thermally and structurally improved 3D-printed CB/PA12 construct components using SLS.

### 6.5. Binder Jetting

**Figure 25:**
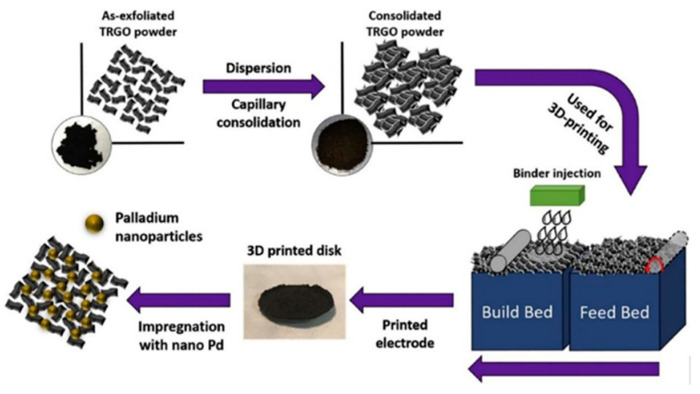
Binder jetting process (adapted with permission from reference [64]).

The common name for binder jetting is three-dimensional printing (3DP), which was introduced by a MIT (Massachusetts Institute of Technology) researcher and used a modified version of inkjet printing. Binder jetting (BJ) uses an inkjet printer head to place the binder into a powder bed, and it is a cold process that does not involve a heating element to bind the material (Figure 25). BJ is an AM technology in which powdered material is placed down sequentially and selectively printed by an ink binder to produce a 3D model [64]. The technology does not require heat, thus, making it is an energy-efficient process, and only a small portion of the part material is delivered through the print head [65].

**Figure 26:**
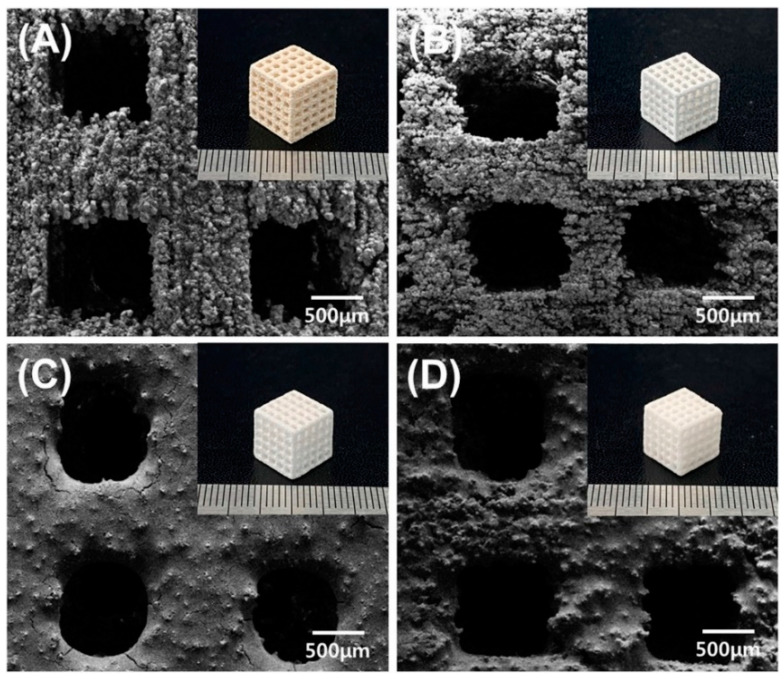
FESEM and (inset) optical images of the surface of the 3D printedscaffolds: (**A**) CSH; (**B**) BCP; (**C**) BCP/d-PCL; (**D**) BCP/m-PCL scaffolds (adapted with permission from reference [66]).

Because of their good mechanical characteristics and biocompatibility for hard tissue engineering applications, Ahn et al. [66] intensively explored biodegradable composite scaffolds. Three-dimensional printing technologies have sparked a lot of interest in the tissue engineering field because of their ability to be customized for tissues repair. Biodegradable polymer-based composite scaffolds with high ceramic loadings were created using the BJ approach in conjunction with capillary rise infiltration. A calcium sulfate hemihydrate (CSH) scaffold was produced via BJ-based 3D printing. Following that, hydrothermal treatment and heat treatment were used to convert CSH into biphasic calcium phosphate (BCP). The generated BCP scaffold was infiltrated with melted polycaprolactone (PCL).Then, BCP was fully distributed in the PCL matrix, with an estimated PCL loading of more than 40% in the BCP matrix. The PCL/BCP composite scaffold exhibited the greatest compressive strength, modulus, and toughness. Furthermore, a stable composite surface enhances early cell responses and pre-osteoblast cell proliferation and differentiation. Figure 26 shows the FESEM and optical images of the surface of the 3D-printed scaffolds.

Many items utilized in a variety of applications, including as ornamental components, prototypes, foundry molds, bone implants, and others, were created using the binder jetting (BJ) method, according to prior research. This approach includes powder deposition to produce the layers, binder application, and post-processing to improve mechanical properties. Fibers may be used with standard raw material powder to create stronger composite components. Because of their low cost, excellent strength, and lack of toxicity, sisal fibers are regarded as potential reinforcement in composites. Coelho et al. [67] utilized BJ to make gypsum–sisal fiber components and looked at the impact of several production factors, including fiber presence, printing orientation, and post-processing. The fibers were shown to improve the mechanical strength of the penetrated regions while causing a loss of strength in the green parts. The loss of mechanical strength caused by the fiber’s increased porosity during the printing process was connected to a loss of mechanical strength; nevertheless, the increased porosity contributed to more successful infiltration post-processing.

**Figure 27:**
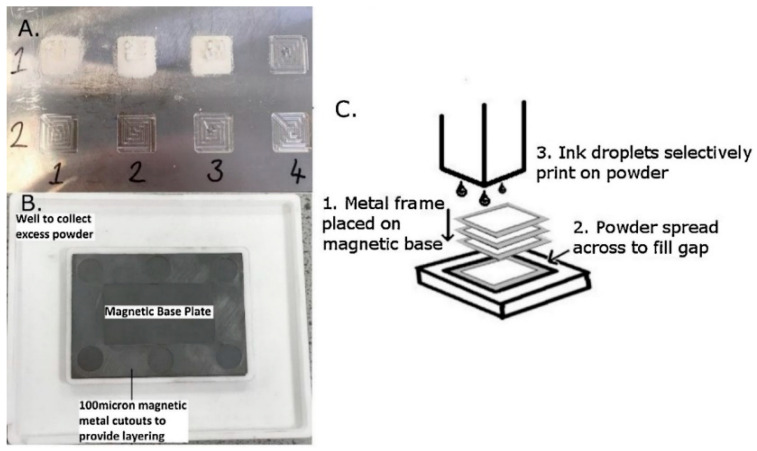
(**A**) A 2D recessed plate for testing powder and ink interactions; (**B**) a 3D powder stacking process; (**C**) an experimental graphic of a 3D layering system in use with processes 1, 2, and 3 cycled until finished (adapted with permission from reference [68]).

Holland et al. [68] utilized a micro-scale powder layering machine under an ink jet printer to examine experimental powders before generating amounts normally used in widely viable binder jetting devices. Powders consisting primarily of ball-milled, amorphous cellulose were efficiently used to construct 3D structures by inducing selective recrystallization when interacting polysaccharides were present in ink (xanthan gum) and as a percentage of the powder component (glucomannan). Because these components are classified as dietary fiber, they may be utilized to make low-calorie 3D-printed food designs for use in food products. Figure 27 depicts a 2D recessed plate for testing powder and ink interaction, as well as a 3D powder layering mechanism, whereas in Figure 27C, a schematic of an experimental 3D layering system in use is depicted.

**Figure 28:**
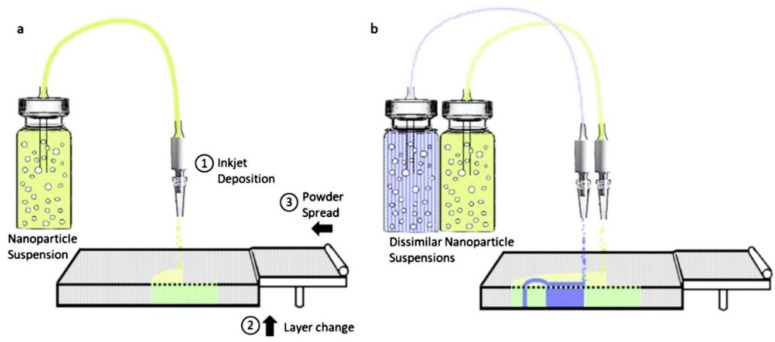
(**a**) The proposed BJ approach to fabricate nanocomposites; (**b**) switching between dissimilar nanoparticle inks produces multimaterial and functionally graded nanocomposites (adapted with permission from reference [69]).

Current AM methods, according to Shen et al. [69], can produce components with complicated shapes utilizing a variety of plastic, metal, and ceramic materials. Integrated technological improvements to print different materials inside the same component are still restricted. Bonding different components necessitate additional processing and result in surfaces with high-stress concentrations. A new binder jetting technique for single-print multimaterial and functionally graded component manufacturing has been described [69]. In the binder, a nanoparticle ink is deposited. Nanocomposites are made up of ceramic, polymer, or metal particles. The technique (see Figure 28) creates a material with graded conductivity and flexibility by switching nanoparticle inks during printing. A graphene oxide (GO) ink is created and printed onto polyvinyl alcohol (PVOH) powder to illustrate the method. A GO/PVOH composite with excellent conductivity and flexibility is the end result. For supercapacitor applications, the composite shows potential as a high-porosity material.

### 6.6. Material Jetting (MJ)

**Figure 29:**
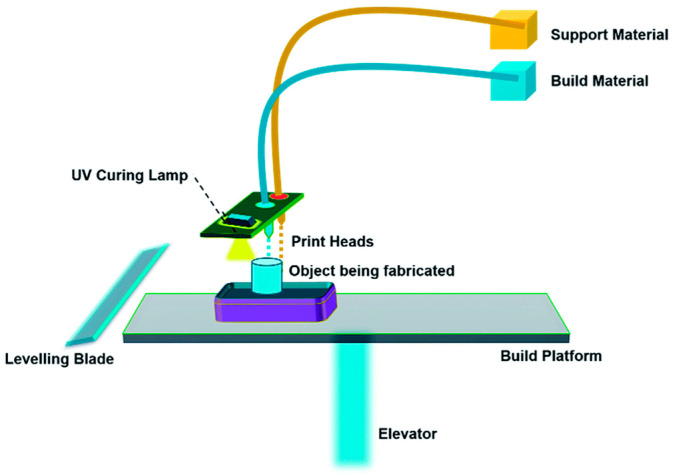
Material jetting process (adapted with permission from reference [72]).

Material jetting (MJ) uses 2D inkjet technology to create objects which are almost the same as the binder jetting (BJ) process. The binder jetting (BJ) process uses a powder-based material and binder, while the MJ process uses liquid photopolymer droplets, which are cured (made solid) with UV light (Figure 29). However, the BJ approach utilizes a binder that was printed onto the powder bed surface to form bonding with the powder and solidified one layer at a time [65]. Furthermore, the MJ approach has gained prominence due to its unique ability to manufacture multimaterial components in a single process using UV curing [70,71]. The capacity to spatially change the mix of hard and soft photopolymer may result in a unique composite behavior with better mechanical characteristics. This property comes in handy for creating soft robots and 4D-printed parts, prompting researchers to look into the geometrical and mechanical properties of the 3D-printed composite structure.

The evolution of flexible electronics and customized therapeutic devices have shifted paradigms owing to 3D-printed carbon nanotubes or graphene-based nanocomposites. The use and characterization of a piezoelectric-pneumatic material-jetting (PPMJ) additive manufacturing technology for printing graphene-based nanocomposites with 3D architectures was addressed by Jabari et al. [70]. The creation of a graphene-silicone ink (MJ-3DG) with a high graphene content (70 wt.%) and its use in the PPMJ method to 3D print a highly conductive graphene-silicone structure is proven in their study. In addition, due to the internal design flexibility of 3D printing and rapid process speed of PPMJ enable the construction of enhanced graphene-based electrical and biomonitoring devices.

To further comprehend the dimensional precision of the PolyJet technique, Tee et al. [71] fabricated microcomposites of 3D-printed polymer composites in order to investigate the effects of reinforced particle arrangements and content. The reinforced particles’ orientations and content both have an influence on the stiffness of a composite when it is crushed. On the one hand, the samples oriented in parallel formation exhibit higher ultimate tensile strength and modulus as compared with the samples oriented at 45° on the construction platform. The composition and orientations of reinforced particles, on the other hand, have a significant impact on the behavior of the samples. Hard particles are discovered to reinforce the component, whereas soft particles act as a fracture starting point.

## 7. Development of 3D Printing to 4D Printing

**Figure 30:**
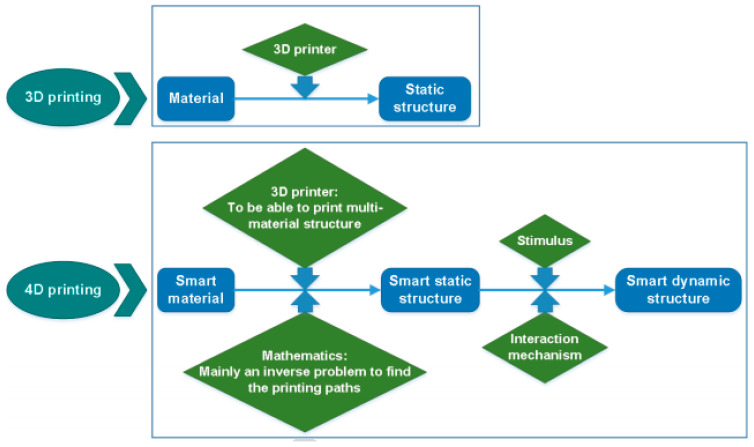
The differences between 3D printing and 4D printing (adapted with permission from reference [73]).

Three-dimesnional printing technology has lasted for almost 30 years now, and the technology has been trending until now. The development of technology has evolved to a higher level with the introduction of 4D printing. There is an addition of one more D than 3D printing that reflected much more value added to the technology. 4D printing is denoted as 3D printing transforming over time by adding the fourth dimension (Figure 30).

## 8. Novel Material Used in 3D Printing Applications

In this study, we concentrated on the most recent developments and applications of new materials in 3D printing. A novel material is a high-tech substance that can be 3D printed for unique use.

### 8.1. Natural Fiber Biocomposite for 3D Printing

Studies on the processing effects and natural fiber properties show an improvement in mechanical properties parallel to the National Policy on Industry 4.0 (Industry 4WRD). Natural fibers have sparked great interest among researchers and industry players for their applications in the military [74], automotive [75,76,77,78,79], industrial [80,81,82,83,84,85,86,87], furniture [88], civil [89,90], and biomedical fields [91]. Applications of NFCs are growing rapidly in numerous engineering fields. Various types of natural fibers have been used as reinforcements in polymer composites, including corn [92], water hyacinth [82], coir [93], ginger [94,95], cotton [96,97], kenaf [98,99,100,101], sugarcane [102,103,104], flax [105], ramie [106], hemp [107], arrowroot [108], kapok [109], sisal [110], wood [111], oil palm [112,113], banana [114], lemongrass [115,116], as well as sugar palm [117,118,119,120,121,122,123,124,125,126,127,128]. Commonly, the most utilized fiber in 3D printing is carbon fiber [129,130] along with glass [131,132,133], Kevlar [134,135,136], or natural fibers [23,137,138]. In addition, there are various hybrid materials that combine plastics with powders to give new color, finish, or extra material properties. Usually for polymers, these materials are typically fabricated from 70% polymer and other 30% natural fiber material. Advanced materials such as micro- and nanofiber isolated from natural fiber have been increasingly utilized in producing 3D filaments. These fibers have been used to reinforce polymers such as polylactic acid (PLA), and polybutylene succinate (PBS) to enhance the mechanical and barrier properties of polymer bionanocomposites.

### 8.2. Digital and Smart Material

**Figure 31:**
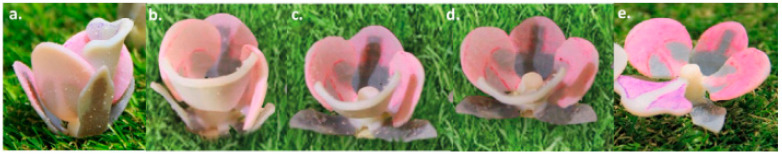
4D printing of smart material (adapted with permission from reference [142]).

The objective of producing a prototype is to ensure that the products are functional, endorse the design, and discuss the concept of the products before being commercially produced. A lot of materials can be 3D printed, such as metals, thermoplastics, and photopolymer [139]. However, the focus in this review is on digital and smart materials, as shown in Figure 31 [140,141]. Figure 31 shows that a 4D-printed flower self-opens upon heat stimulation. The fourth dimension was added to 3D printing to make the object respond against various stimuli.

#### 8.2.1. Digital Material

Advanced composite material consisting of two or three photopolymers in certain microstructures and ratios is called a digital material. The material can produce a functional prototype, including superficial textures, colors, and hardness. For instance, the Stratasys J750 3D printer can load up to six materials simultaneously without changing canisters in a single build process and can incorporate more than 360,000 colors. Hiller and Lipson [143] analyzed the digital materials design for layered manufacturing using material building blocks. They concluded that digital manufacturing of building blocks using 3D printing successfully achieved good precision, thus, showing that the accuracy through this process can be exploited to produce more advanced products [143].

#### 8.2.2. Smart Materials for 4D Printing

Smart material is a material that is able to change its geometry due to external stimuli [144,145,146]. The external stimuli are stated to transform the shape of the material over time slowly. Humidity and heat are examples of external stimuli which may occur. The notion of 4D printing is based on the 3D printing of programmable smart materials, and it has become the most prominent concern in the 3D printing sector, where the fourth dimension is time [147,148].

### 8.3. Ceramic Material

**Figure 32:**
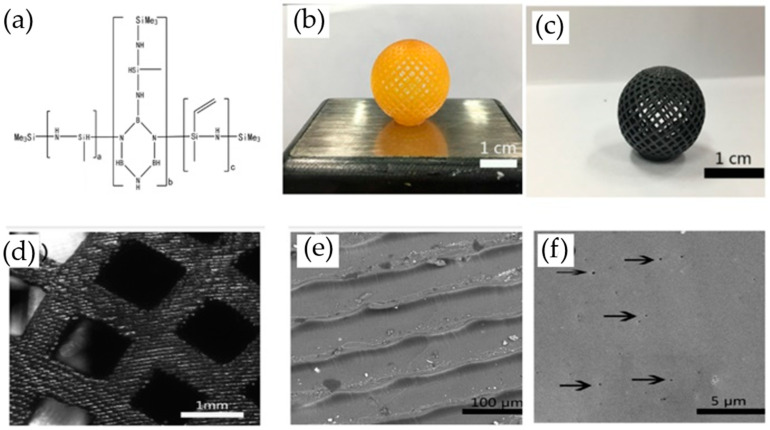
3D printing of polymer-derived ceramics (adapted with permission from reference [150,151]).

**Figure 33:**
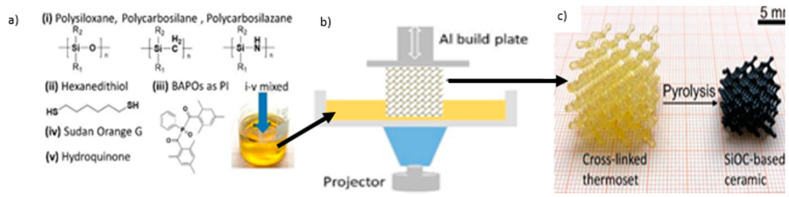
A schematic representation of 3D printed preceramic polymers [151].

Material such as ceramic and concrete cannot be printed using 3D printing due to the fact that the individual powder cannot be fused by applying heat to their melting point (Figure 32 and Figure 33). However, polymers and metals can be fused under heat at their glass transition temperature (T_g_). As comparison with polymer and metal, the melting point of ceramic is extremely higher than both materials, and therefore the process of ceramic 3D printing is very difficult to be carried out [149].

The mechanical properties of ceramic made from the 3D printing process are comparable to traditionally fabricated ceramic parts. The current 3D printing process has also yielded ceramic parts without any large pores through the optimization of 3D printing parameters. There is also the possibility of producing ceramic parts without pores by combining colloidal processing techniques with the incorporation of extra densification steps after the 3D printing process [149].

### 8.4. Electronic Material

In recent decades, electronic materials have achieved a significant improvement in the field of 3D printing [152,153]. Some people are questioning whether the technology is suitable for using in the production of in a large volume of electronics [154].

Currently, available technology has enabled manufacturers to produce functional electronics, for instance, inductors, resistors, capacitors, and antennae, in a one-step without any post-processing [155]. Electronic material is commonly 3D printed using inkjet printing and aerosol jet printing, whereby the technology is using a nozzle for printing, thus, it can avoid direct contact with the electronic material. Kim, Lee, Jeong, and Moon [156] used self-synthesized silver ink to fabricate a thin transistor film that was flexible on plastic.

Another researcher also managed to reveal a way to print resistors on a plastic substrate using a conducting polymer. The result showed a higher resistance value was achievable with high repeatability [157].

### 8.5. 3D Printing of Fiber-Reinforced Polymer Composites

Fiber-reinforced composites have been proven to improve the properties of 3D-printed components with a polymer matrix. The common problems, such as void content and fiber orientation of composites need to be taken into consideration in 3D-printed composites. The 3D printing industry will benefit greatly from the reinforcement.

### 8.6. Aerospace Application

The aerospace industry has benefited from 3D printing technology since it was introduced. The technology offers a product that has complex engineered geometries with a shorter production time. Moreover, products from 3D printing technology are very suitable for the aerospace industry since they have several advantages such as lightweight, able to withstand high temperature, long useful live, and enable low production volume [158]. Kestilä et al. [159] investigated the benefits of combining plastic 3D printing and atomic layer deposition (ALD) coating in producing a propulsion component with the aim of improving its structural integrity, propellant flow performance, outgassing properties, and thermal resistance. The findings revealed that the coating used may help to reduce outgassing at higher temperatures, but the evidence was still inconclusive, and more research is needed.

### 8.7. Medical Application

Current and future developments in the AM processes, devices, and materials would allow for increased applications in the medical and dental area. Three-dimensional printing technologies such as SLM, SLA, FDM, and DLP have been used in dentistry. Anatomical models produced using 3D printing have allowed surgeons to have an overview of a complex structure before surgery is carried out by referring to the 3D physical model of the skull or other structures [160]. Other AM processes and application areas include powder bed fusion of metal implants [141], additive manufacturing of medical instruments [142], biomaterials in medical additive manufacturing [143], and medical phantoms and regenerated tissue and organ applications [144].

### 8.8. Automotive Application

The automotive industry is confronted with new challenges where new design trends and technological deployments from research push companies to develop new models and facelifts in the short term, necessitating the development of new tools or the reshaping of existing tools. In the automotive industry, additive manufacturing has been used to tool up a stamping process for the production of body panels and brake pedals [161]. The findings led to the conclusion that metal additive manufacturing provided stamping tools with excellent performance and a significant reduction in time-to-tooling [162]. Given the rigidity of the milestones imposed on the automotive industry, it could be a key decision factor in enabling on-time tool production [163,164,165,166].

### 8.9. Consumer Product

The major paradigm in the era of democratized production is a prosumer reconfiguration of consumer products. By allowing consumers to modify and extend products, a 3D printing platform for prosumer reconfiguration that connects consumers and producers in the material domain can free consumers from being locked into a producer’s product line. This prosumer-oriented ecosystem of consumer products will discourage redundant functionality proliferation and encourage feature divergence to meet the creative needs of individual consumers [167]. Dahake et al. [168] reviewed the application of medical rapid prototyping (MRP)-assisted customized surgical guides (CSGs). The study concluded that the MRP-assisted CSGs improved surgical efficiency by making surgery much faster, more precise, and less expensive than any other technique. The use of AM in the repair supply chain reduces the number of products that must be reimbursed to customers as a result of lengthy repairs, improves the repair shop’s repair statistics, and reduces the number of items held in stock [169].

## 9. Conclusions and Future Remarks

The emergence and development of 3D printing technology, as well as accrescent demand for high-end products and smart materials, have transformed the landscape of the manufacturing industry. The industry has evolved significantly over the past 30 years, and a wide range of industries have already adopted 3D printing technology. The technology has created a new horizon in the manufacturing industry, in which products that could not be possible with other manufacturing processes have become a reality. In addition, the correct terminology must be used in additive manufacturing to describe the general terms, process categories (general processing, data processing, material processing), applications, and properties. Thus, the general terms and definitions for additive manufacturing were established according to ISO/ASTM 52900, as discussed in this manuscript. The International Standard establishes and defines terms used in additive manufacturing (AM) technology, which are applied to the additive shaping principle, and thereby build physical 3D geometries by successive addition of material.

Cost-effective, flexible design, and rapid prototyping are the main advantages of 3D printing. The 3D printing technology is also an environmentally friendly process where it involves manufacturing layer-by-layer products that can reduce wastes. The technology is sustainable since the waste is reduced, and the material can be reused and recycled to produce other 3D-printed products.

The lead time to produce products can be significantly shortened since the processes involved have been simplified. The assembly parts of products that involved geometrically complex parts can be reduced, and the production has become faster and more accurate. The 3D printing technology has approached maturity and has driven the growth of the industry by unlocking potential new business opportunities and, at the same time, by supporting mass customization. Current 3D printing technology can still be further enhanced, for instance, using alternative scanners, new 3D printers, and a variety of base materials. In conclusion, 3D printing technologies have been accepted by most industries and will become a revolutionizing manufacturing process in various industrial areas in the near future.

## Figures and Tables

**Table 1:** Advantages and disadvantages of 3D printing.

Advantages	Disadvantages
Flexible design	Limited materials
Rapid prototyping	Restricted build size
Print on demand	Post processing
Strong and lightweight parts	Large volumes
Fast design and production	Part structure
Minimizing waste	Reduction in manufacturing jobs
Cost effective	Design inaccuracies
Ease of access	Copyright issues
Environmentally friendly	
Advanced applications	

**Table 2:** Terms and definition of additive manufacturing in ISO/ASTM 52900.

Term	Definition
General terms	
3D printer	Machine Used for 3D Printing
Additive manufacturing (AM)	Process of joining materials to make parts from 3D model data, usually layer upon layer, as opposed to subtractive and formative manufacturing methodologies
Additive system	Additive manufacturing equipment
AM machine	Section of the additive manufacturing system including hardware, machine control software, required setup software, and peripheral accessories necessary to complete a build cycle for producing parts
AM machine user	Operator of or entity using an AM machine
AM system user	Operator of or entity using an entire additive manufacturing system or any component of an additive system
Front	<of a machine; unless otherwise designated by the machine builder> Side of the machine that the operator faces to access the user interface or primary viewing window, or both
Material supplier	Provider of material/feedstock to be processed in an additive manufacturing system
Multistep process	Type of additive manufacturing process in which parts are fabricated in two or more operations where the first operation typically provides the basic geometric shape and the following operation(s) consolidate the part to the fundamental properties of the intended material (metallic, ceramic, polymer, or composite)
Single-step process	Type of additive manufacturing process in which parts are fabricated in a single operation where the basic geometric shape and basic material properties of the intended product are achieved simultaneously
Process Categories	
Binder jetting	Additive manufacturing process in which a liquid bonding agent is selectively deposited to join powder materials
Directed energy deposition	Additive manufacturing process in which focused thermal energy is used to fuse materials by melting as they are being deposited
Material extrusion	Additive manufacturing process in which material is selectively dispensed through a nozzle or orifice
Material jetting	Additive manufacturing process in which droplets of build material are selectively deposited
Powder bed fusion	Additive manufacturing process in which thermal energy selectively fuses regions of a powder bed
Sheet lamination	Additive manufacturing process in which sheets of material are bonded to form a part
Vat photopolymerization	Additive manufacturing process in which liquid photopolymer in a vat is selectively cured by light-activated polymerization
Processing: General	
3D printing	Fabrication of objects through the deposition of a material using a print head, nozzle, or another printer technology
Build chamber	Enclosed location within the additive manufacturing system where the parts are fabricated
Build cycle	Single process cycle in which one or more components are built up in layers in the process chamber of the additive manufacturing system
Build envelope	Largest external dimensions of the x-, y-, and z-axes within the build space where parts can be fabricated
Build platform	<of a machine> Base which provides a surface, upon which the building of the part/s is started and supported throughout the build process
Build space	Location where it is possible for parts to be fabricated, typically within the build chamber or on a build platform
Build surface	Area where material is added, normally on the last deposited layer which becomes the foundation upon which the next layer is formed
Build volume	Total usable volume available in the machine for building parts
Feed region	<in powder bed fusion> Location/s in the machine where feedstock is stored and from which a portion of the feedstock is repeatedly conveyed to the powder bed during the build cycle
Layer	<matter> Material laid out, or spread, to create a surface
Machine coordinate system	Three-dimensional coordinate system as defined by a fixed point on the build platform with the three principal axes labeled x-, y-, and z-, with rotary axis about each of these axis labeled A, B, and C, respectively, where the angles between x-, y-, and z- can be Cartesian or defined by the machine manufacturer
Manufacturing lot	Set of manufactured parts having commonality between feedstock, production run, additive manufacturing system, and post-processing steps (if required) as recorded on a single manufacturing work order
Origin	Zero point (0, 0, 0) <when using x-, y-, and z-coordinates> designated as the universal reference point at which the three primary axes in a coordinate system intersect
Build origin	Origin most commonly located at the centre of the build platform and fixed on the build facing surface, but could be defined otherwise by the build setup
Machine origin	Machine home, machine zero point, origin as defined by the machine manufacturer
Overflow region	<in powder bed fusion systems> Location/s in the machine where excess powder is stored during a build cycle
Part location	Location of the part within the build volume
Process parameters	Set of operating parameters and system settings used during a build cycle
Production run	All parts produced in one build cycle or sequential series of build cycles using the same feedstock batch and process conditions
System setup	Configuration of the additive manufacturing system for a build
*x*-Axis	<of a machine; unless otherwise designated by the machine builder> Axis in the machine coordinate system that runs parallel to the front of the machine and perpendicular to the *y*-axis and *z*-axis
*y*-Axis	<of a machine; unless otherwise designated by the machine builder> Axis in the machine coordinate system that runs perpendicular to the *z*-axis and *x*-axis
*z*-Axis	<of a machine; unless otherwise designated by the machine builder> Axis in the machine coordinate system that run perpendicular to the *x*-axis and *y*-axis
Processing: Data	
3D scanning	Method of acquiring the shape and size of an object as a 3-dimensional representation by recording x, y, z coordinates on the object’s surface and through software the collection of points is converted into digital data
Additive Manufacturing File Format (AMF)	File format for communicating additive manufacturing model data including a description of the 3D surface geometry with native support for colour, materials, lattices, textures, constellations and metadata
Bounding box	<of a part> orthogonally oriented minimum perimeter cuboid that can span the maximum extents of the points on the surface of a 3D part
Arbitrarily oriented bounding box	<of a part> bounding box calculated without any constraints on the resulting orientation of the box
Machine bounding box	<of a part> bounding box for which the surfaces are parallel to the machine coordinate system
Master bounding box	bounding box which encloses all of the parts in a single build
Extensible markup language (XML)	Standard from the WorldWideWeb Consortium (W3C) that provides for tagging of information content within documents offering a means for representation of content in a format that is both human and machine readable
Facet	Typically a three- or four-sided polygon that represents an element of a 3D polygonal mesh surface or model
geometric centre	<of a bounding box> Location at the arithmetic middle of the bounding box of the part
IGES	Initial graphics exchange specification, platform neutral CAD data exchange format intended for exchange of product geometry and geometry annotation information
Initial build orientation	<of a part> Orientation of the part as it is first placed in the build volume
Nesting	Situation when parts are made in one build cycle and are located such that their bounding boxes, arbitrarily oriented or otherwise, will overlap
PDES	Product data exchange specification or product data exchange using STEP
Part reorientation	Rotation around the geometric centre of the part’s bounding box from the specified initial build orientation of that part
STEP	Standard for the exchange of product model data
STL	File format for model data describing the surface geometry of an object as a tessellation of triangles used to communicate 3D geometries to machines in order to build physical parts
Surface model	Mathematical or digital representation of an object as a set of planar or curved surfaces, or both, that can, but does not necessarily have to, represent a closed volume
Processing: Material	
Curing	Chemical process which results in the ultimate properties of a finish or other material
Feedstock	Source material/starting material/base material/original material bulk raw material supplied to the additive manufacturing building process
Fusion	Act of uniting two or more units of material into a single unit of material
Laser sintering (LS)	Powder bed fusion process used to produce objects from powdered materials using one or more lasers to selectively fuse or melt the particles at the surface, layer upon layer, in an enclosed chamber
Part cake	<in a powder bed fusion process that uses a heated build chamber> Lightly bound powder surrounding the fabricated parts at the end of a build cycle
Post-processing	<one or more> Process steps taken after the completion of an additive manufacturing build cycle in order to achieve the desired properties in the final product
Powder batch	Powder used as feedstock which could be used powder, virgin powder or a blend of the two
Powder bed	Part bed, build area in an additive manufacturing system in which feedstock is deposited and selectively fused by means of a heat source or bonded by means of an adhesive to build up parts
Powder blend	Quantity of powder made by thoroughly intermingling powders originating from one or several powder lots of the same nominal composition
Powder lot	Quantity of powder produced under traceable, controlled conditions, from a single powder manufacturing process cycle
Used powder	Powder that has been supplied as feedstock to an AM machine during at least one previous build cycle
Virgin powder	Unused powder from a single powder lot
Applications	
Part	Joined material forming a functional element that could constitute all or a section of an intended product
Prototype	Physical representation of all or a component of a product that, although limited in some way, can be used for analysis, design and evaluation
Rapid prototyping	<in additive manufacturing> Application of additive manufacturing intended for reducing the time needed for producing prototypes
Rapid tooling	<in additive manufacturing> Application of additive manufacturing intended for the production of tools or tooling components with reduced lead times as compared to conventional tooling manufacturing
Properties	
Accuracy	Closeness of agreement between an individual result and an accepted reference value
As built	refers to the state of parts made by an additive process before any post processing, besides, if necessary, the removal from a build platform as well as the removal of support and/or unprocessed feedstock
Fully dense	State in which the material of the fabricated part is without significant content of voids
Near net shape	Condition where the components require little post-processing to meet dimensional tolerance
Porosity	<property> Presence of small voids in a part making it less than fully dense
Repeatability	Degree of alignment of two or more measurements of the same property using the same equipment and in the same environment

## Data Availability

Not applicable.
